# King rail (*Rallus elegans*) home range and microhabitat characteristics in western Lake Erie coastal marshes

**DOI:** 10.1002/ece3.10043

**Published:** 2023-04-26

**Authors:** Dustin E. Brewer, Thomas M. Gehring, Madeline M. Garcia, Brendan T. Shirkey, John W. Simpson, Auriel M. V. Fournier

**Affiliations:** ^1^ Department of Biology, Institute for Great Lakes Research Central Michigan University Mt. Pleasant Michigan USA; ^2^ Winous Point Marsh Conservancy Port Clinton Ohio USA; ^3^ Forbes Biological Station–Bellrose Waterfowl Research Center, Illinois Natural History Survey, Prairie Research Institute University of Illinois at Urbana‐Champaign Havana Illinois USA

**Keywords:** Great Lakes, marsh, radio telemetry, *Rallus elegans*, secretive marsh birds

## Abstract

The king rail (*Rallus elegans*) is a secretive marsh bird that is threatened or endangered in eight of nine states and provinces in the Laurentian Great Lakes (Great Lakes) region. Available survey data suggests that this species has undergone population declines across this region and these are believed to have been driven by habitat loss and degradation. An improved understanding of the amount and type of habitat king rails require during the breeding season at sites within the Great Lakes region would inform and improve progress toward conservation goals. During 2019–2021, we caught and radio‐tagged 14 king rails in northwestern Ohio and southeastern Michigan within impounded coastal wetlands of western Lake Erie. We used radio telemetry to identify breeding season (May–August) home‐range characteristics and third order habitat selection within home ranges (hereafter microhabitat). For the birds whose home range stabilized (*N* = 10), we found a mean home‐range size of 8.8 ha (±1.63 [SE]; range = 1.9 to 15.8). We generated a classification tree to determine which habitat characteristics were associated with king rail presence within home ranges in our study. We found that vegetative density within home ranges was particularly associated with king rail presence. *Phragmites australis* was also associated with king rail presence, despite its invasiveness and negative ecological impacts in the region, and could be selectively maintained to benefit king rails. Our results suggest that managers may be able to provide microhabitat for king rails by maintaining water depths of 6 to 17 cm and by promoting native, robust vegetation in the genera *Carex* and *Juncus*. Our findings could help inform wetland managers and conservation planners in the Great Lakes region, particularly in western Lake Erie coastal marshes, of patch sizes, water depths, plant communities, and vegetative structure preferred by king rails.

## Graphical abstract

We studied microhabitat and home‐range characteristics of the king rail, a secretive marsh bird of conservation concern, in the western Lake Erie region. Our results suggest that king rails in the region have mean home‐range sizes of 8.8 ha (±1.63 [SE]; range = 1.9 to 15.8) and utilize water depths of 6 to 17 cm within dense vegetation. These findings could help wetland managers to create and maintain king rail habitat.

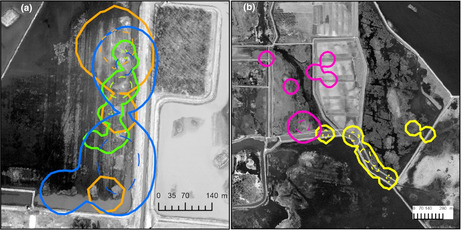

## 1 INTRODUCTION

Human activity has severely altered the spatial extent of wetlands. Such alteration has occurred extensively since the mid‐1800s in the 8 U.S. states (IL, IN, MI, MN, NY, OH, PA, WI) and Canadian province (ON) that border the Laurentian Great Lakes (hereafter Great Lakes; Iverson, 1988, Simpson et al., 1994, Radeloff et al., 1999). During this period, and similar to global trends (Davidson, 2014), there has been a reduction in wetland cover by ~50% or more in each of the U.S. Great Lakes states, of which three had wetland area reduced by ≥85% (Dahl, 2011; Dahl & Allord, 1996). Extensive wetland loss has also occurred in southern Ontario, CAN, where wetland losses have exceeded 70% (Ducks Unlimited Canada, 2010; Penfound & Vaz, 2022). Before European colonization, the Great Lakes region was covered by a relatively large proportion of wetland land cover due to glacial history. For example, approximately 20% of Ohio's landcover in 1700 was wetland (Fretwell, 1996)—with a large proportion of this area in the northwest along the coast of Lake Erie (Kaatz, 1955)—though only about 2% of the state's landcover is now wetland (Fretwell, 1996). Reductions in the extent of suitable habitat affects avian biodiversity (Herkert, 1994; Rittenhouse et al., 2012; Tozer, 2016). Given the extreme decline in wetland area in the Great Lakes region since the mid‐1800s, we assume that most wetland‐dependent bird species—though perhaps not some waterfowl species—have consequently declined in abundance (Rosenberg et al., 2019).

The king rail (*Rallus elegans*) exemplifies a wetland‐dependent species which has, since the mid‐1800s, declined in abundance throughout much of its range (Pickens & Meanley, 2020). However, the degree of population declines during most of this period is unclear due to limited monitoring. Breeding Bird Survey (BBS) data, albeit not ideal for monitoring marsh birds (Conway & Gibbs, 2005), indicate a decline in range‐wide king rail abundance by ~4% per year between 1966 and 2019 (Pardieck et al., 2020). There are numerous examples of extirpation or severe population decline especially at northern localities within the king rail's migratory range (Cooper, 2008; Pickens & Meanley, 2020), where breeding occurs but overwintering does not. King rail declines in the southern U.S. (Budd & Krementz, 2011) have likely contributed to population declines in the migratory range given that individuals migrate north in some years but not others (Kane et al., 2019). King rails are a game species in some southern states, like Louisiana, which could also affect northern populations (Kane et al., 2019). The king rail is listed as a species of greatest conservation need in 30 U.S. states and, of these, is threatened or endangered in 12 states as well as in Ontario, CAN—which are all in the migratory range (Cooper, 2008; Kane et al., 2019; Kraus, 2016). The Upper Mississippi River and Great Lakes (UMGL) Joint Venture—which includes all of Michigan, Indiana, Wisconsin, and parts of Ohio, Illinois, Missouri, Kansas, Nebraska, Iowa, and Minnesota—has identified the king rail as a focal species due to its conservation need and potential to represent a segment of wetland biodiversity (Soulliere et al., 2007). The UMGL Joint Venture is a partnership of diverse organizations that work together to accomplish conservation goals such as increasing king rail populations within joint venture boundaries, where declines have been particularly severe (Cooper, 2008).

King rail conservation efforts across the migratory range are ongoing. For example, the UMGL Joint Venture's goal regarding king rails is to double breeding populations within its jurisdictional boundaries, where in 2007 <400 individuals were estimated to occur during the breeding season (Soulliere et al., 2007). Occupancy and trend estimates for the region, however, are lacking due to the paucity of king rail detections during marsh bird surveys (Monfils et al., 2020). To increase the population of king rails in the Great Lakes region, and to avoid an extinction vortex (e.g., Kampichler et al., 2010), appropriate habitat likely needs to be restored and/or maintained therein (Soulliere et al., 2007). However, perhaps due to the low abundance (Bolenbaugh et al., 2012) and secretive nature of king rails in the Great Lakes region, there is limited knowledge regarding what habitat or amount of space individuals require and to what degree changes in habitat availability and composition have contributed to population decline. Consequently, wetland managers, biologists, and policymakers have lacked information required to help accomplish regional king rail conservation goals.

Although the landscape scale (i.e., second order habitat selection; Johnson, 1980) is an important indicator of king rail home‐range selection (sensu Powell, 2000), previous studies in the Great Lakes region (Darrah & Krementz, 2009; Kane, 2020) did not involve individuals that were tracked to the specific points where they occurred naturally. Therefore, these studies were limited regarding ability to infer about third‐order habitat selection (Johnson, 1980), that is, habitat selected within a home range, which we refer to herein as microhabitat. Multiple scales are important for determining habitat associations (e.g., Kellner et al., 2016) and must be considered to successfully manage king rails in the Great Lakes region. Radio telemetry has been used to study king rail home‐range characteristics and microhabitat use outside of the Great Lakes region (Kolts & McRae, 2017; Pickens & King, 2013). However, the results of these studies may not be applicable due to documented geographic variability in habitat use by king rails (Meanley, 1969).

Knowledge of king rail breeding season home range and microhabitat characteristics in the Great Lakes region could advance the ability of conservation planners and wetland managers to effectively deliver habitat for king rails. Some areas within the Great Lakes region—like western Lake Erie coastal marshes—appear to support relatively large king rail populations compared to other areas therein (Hansen, 2019; Soulliere et al., 2007) and so are ideal for studies that focus on capturing individuals. King rail space use and microhabitat inferences drawn from such areas could potentially apply to much of the Great Lakes region, especially coastal regions. Consequently, our objectives within western Lake Erie coastal marshes were: (1) to determine king rail breeding season home‐range characteristics including size/overlap and (2) to describe the microhabitat conditions selected by king rails during the breeding season.

## 2 METHODS

**FIGURE 1:**
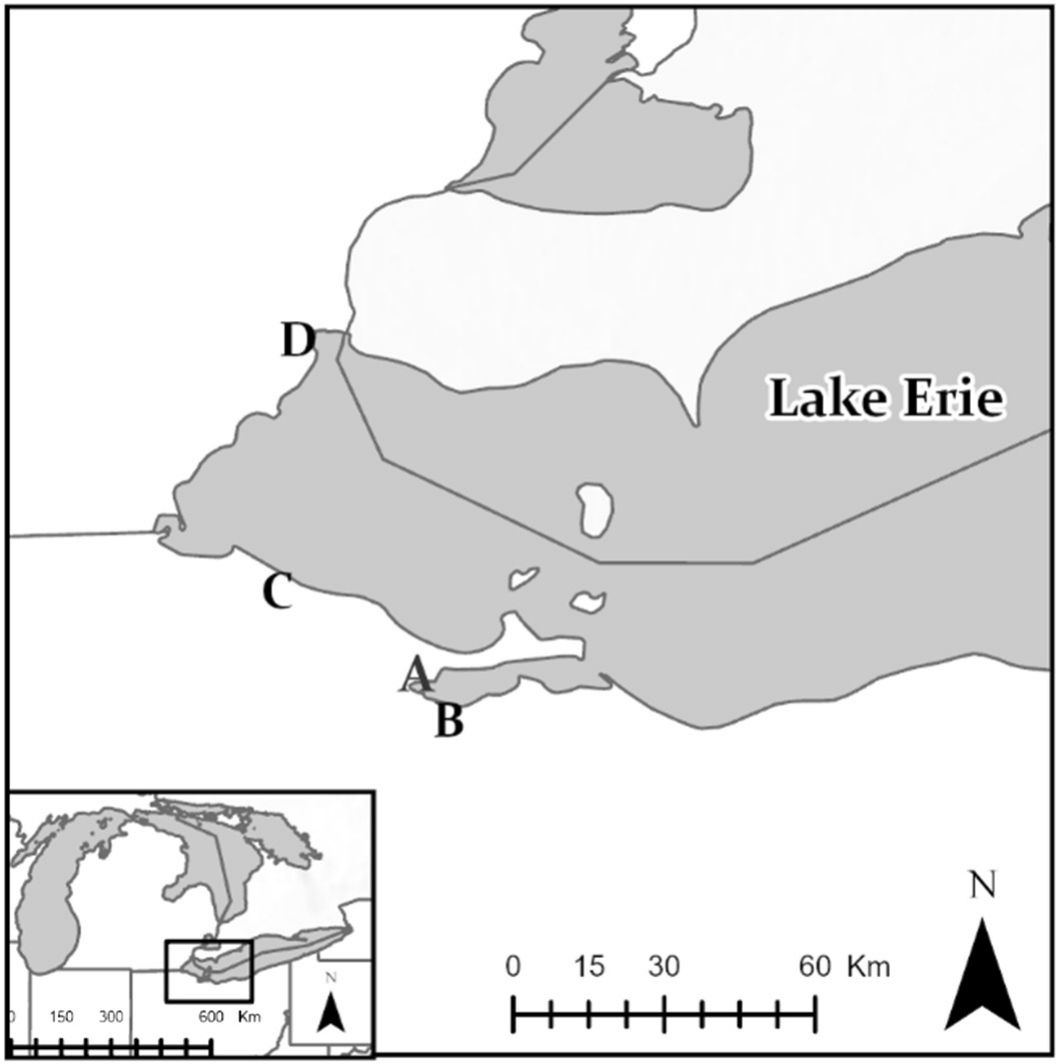
Our study area (in Michigan and Ohio, USA), with sites where we caught king rails from May 2019 to June 2021 labeled. A = Winous Point Marsh Conservancy; B = Pickerel Creek Wildlife Area; C = Ottawa National Wildlife Refuge; D = Pointe Mouillee State Game Area. We quantified home‐range size and microhabitat use at sites A, B, and D. The bird caught at site C left the area before its home range stabilized.

We conducted this study in western Lake Erie coastal marshes of northwestern Ohio and southeastern Michigan, USA, between the latitudes of 41.40° and 42.04° and the longitudes of −82.22° and −82.92° (Figure 1). This was a temperate region near the northernmost part of the range of king rails. Average high temperatures during the study period ranged from 18.3 to 27.8°C and low temperatures ranged from 7.7 to 17.8°C. Wetlands within the study area tended (estimated 95% of wetland area) to be impounded and managed for waterfowl. These impounded marshes were dominated by herbaceous emergent vegetation, including the genera *Typha*, *Phragmites*, and *Carex*, with areas of open water interspersed. Water depths within impoundments varied based on management strategy. Generally, water depths were >0.6 m throughout the summer to deter invasive species and to promote native aquatic vegetation or brought to zero to promote growth of seed‐rich moist‐soil species, which provide high‐energy food for waterfowl in the fall (Smith et al., 1989). We trapped king rails on both public (Pickerel Creek Wildlife Area, OH; Ottawa National Wildlife Refuge, OH; Pointe Mouillee State Game Area, MI) and private (Winous Point Marsh Conservancy, OH) property (Figure 1).

We trapped king rails from early May through late June between 2019 and 2021. We selected trapping sites based on past sightings and captures during prior years and located birds using call‐broadcast techniques (Shirkey et al., 2017). We captured king rails using whoosh nets and walk‐in traps (Shirkey et al., 2017). Upon capture, we attached VHF radio transmitters (ATS – Isanti, Minnesota; model A1050; weight: 2.4 g; frequency range: 164.3 to 165.4 MHz) to king rails using leg‐loop harnesses (Rappole & Tipton, 1991) made of 0.7 mm diameter stretch magic cord (Stretch Magic Inc – Sonoma California). We collected blood from the ulnar vein (~100 microliters), to allow genetic sexing of individuals, and recorded the following morphometric measurements: mass, wing chord, bill length, tarsus length, and tail length. We recorded these measurements to aid with sex identification and the blood samples were shipped off‐site for analysis which followed the same procedure described by Kolts and McRae (2017). We released birds at the location where they were captured within 1 hour of removal from a trap.

### 2.1 Tracking

We began tracking king rails using radio telemetry (White & Garrott, 1990) >24 h after attaching radio transmitters to allow individuals time to resume normal activity. Homing consisted of using handheld radio telemetry equipment (ATS – Isanti, Minnesota; R2000 and R4000, 3‐element folding Yagi antenna) to track individuals to where we assumed they were naturally occurring and then we used a GPS to mark coordinates (similar to Pickens & King, 2013). We slowly approached focal individuals so that they were minimally disturbed before homing points were marked. We triangulated or biangulated the location of individuals when approximately 20 m away and approached closer to estimate the specific location where they occurred. We marked each location >24 h after the previous location to maximize both independence between points and frequency of sampling. We typically marked locations for individuals 3 times per week during a span of 14 weeks (12 May to 18 August). During each week, our goal was to mark locations during three different time periods on separate days: morning (within 4 h after sunrise), mid‐day (within 2 h of noon), and evening (within 4 h before sunset). Each week, we randomly determined the order of these surveys for each individual and adjusted only if there were logistical constraints.

### 2.2 Home‐range analysis

We used homing points marked throughout the breeding season to estimate the size of home ranges and the size of core areas (i.e., regions of concentrated use). We excluded all homing points that did not occur within 500 m of ≥4 other homing points, which excluded rare instances when individuals appeared to be demonstrating exploratory behavior, based on home‐range sizes described by Pickens and King (2013) and Kolts and McRae (2017). We only calculated home ranges for individuals whose home‐range sizes reached a stable asymptote as determined by visual analysis of plots. The minimum number of homing points used to construct a home range was 14, which was the same minimum used by Kolts and McRae (2017). The maximum number of homing points for an individual was 36.

We constructed 95% kernel density home ranges (hereafter home ranges) and 50% kernel density core use areas (hereafter core areas) using the adehabitatHR package (Calenge, 2006) in R v. 4.0.3 (R Core Team, 2020). This kernel density approach can achieve low bias in home‐range estimation despite a moderate degree of autocorrelation (Swihart & Slade, 1997). Regardless, we intended for our sampling approach to avoid autocorrelation given that our habitat analysis approach (see below) required independent observations. Similar to Kie (2013), we used an adaptive approach to select smoothing parameter (*h*) values. For each individual, we began with an *h* determined by least‐squares cross‐validation and superimposed that initial 95% density home range on a satellite image. If necessary, we then iteratively modified *h* to both minimize the number of polygons within the focal home range and to avoid inclusion in the home range of cover types, like upland forest, that were never used by king rails during our study. Finally, we projected each home range onto a map via Google Earth Pro and used the polygon ruler tool to determine the degree to which each home range overlapped with others.

### 2.3 Microhabitat measurement

**FIGURE 2:**
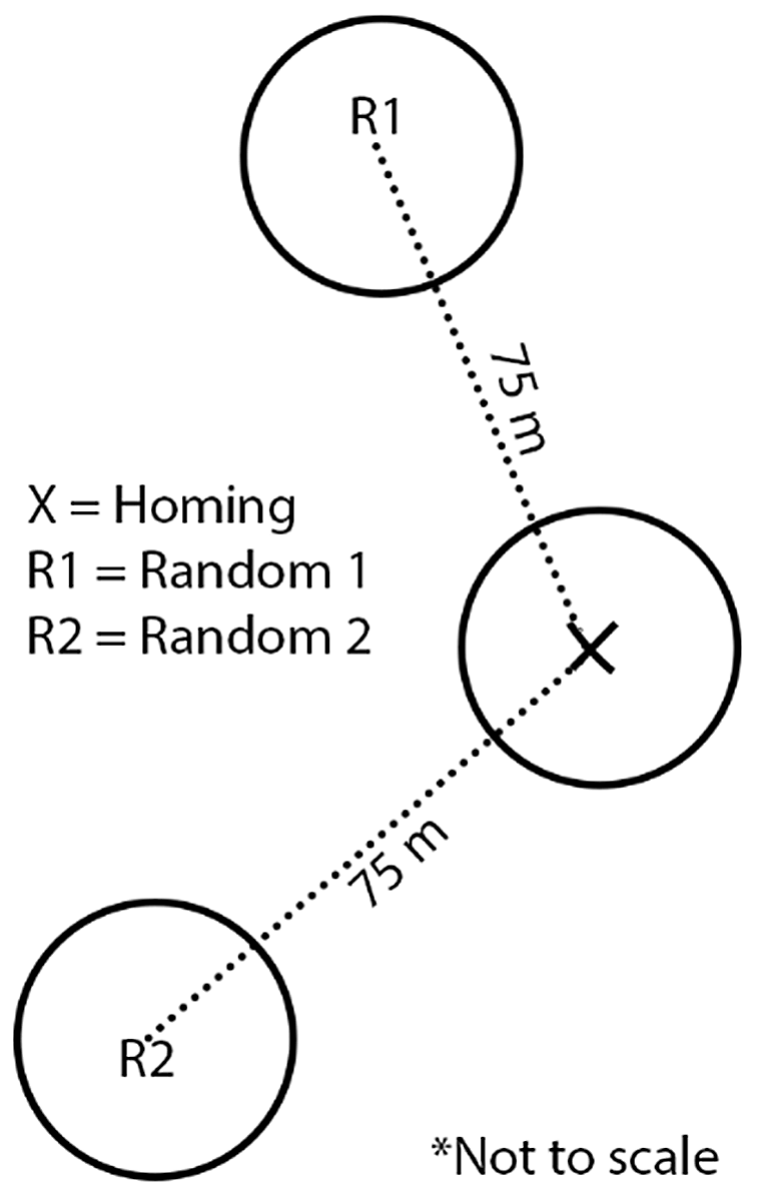
Example of points sampled during microhabitat surveys at and in the vicinity of a homing point during our study in Ohio and Michigan, USA (from May 2019 to August 2021). The homing point (X) represents microhabitat where a king rail was naturally occurring and the random points (R1, R2) are in a random direction from the homing point and represent nonused conditions. Random points were separated by at least 25 degrees.

We conducted three microhabitat surveys at or in the vicinity of each homing location within 72 h of when a homing point was marked. Each of these surveys occurred within a unique 5‐m radius plot to describe local conditions. We conducted one survey at the homing location that represented microhabitat used by the focal individual. We conducted two additional habitat surveys 75 m in random directions from the homing location so that they represented conditions not used by the focal individual at the time of homing (Figure 2). We chose 75 m to maximize likelihood that these random points occurred within the home range but were distant enough from the homing point to represent distinct, within‐home range conditions that were not being actively used at the time the homing point was marked. If we suspected that homing points occurred in the same location during successive days because of nesting behavior, we only conducted one set of microhabitat surveys at or in the vicinity of that location to avoid disturbing a nesting attempt.

We recorded microhabitat measurements that, based on previous research (Darrah & Krementz, 2009; Hengst, 2021; Kolts & McRae, 2017; Pickens & King, 2013), we hypothesized could describe king rail microhabitat selection in our study area. At each point we recorded water depths, proximity to landscape features, plot coverage by landcover category, and additional vegetative characteristics, including density, structure, and plot coverage by plant category (Table 1). The protocol that describes these microhabitat measures in detail, including which species are included in each plant category, is provided (see Appendix A). Also, because it has been suggested that crayfish are an important food source for king rails (Pickens & Meanley, 2020), we estimated crayfish (Cambaridae) relative abundance at homing and random points up to twice per week per radio‐tagged bird during the last 3 weeks of July, when crayfish were commonly observed at our study site. Of the two random points associated with each homing point where trapping occurred, we randomly chose one for crayfish trapping. We recorded the number of crayfish, regardless of species, that were caught in funnel traps (30.5 cm × 25.4 cm × 15.2 cm) baited with dry dogfood and placed within microhabitat plots until the next day, for ≤24 h (Figure 2).

**TABLE 1:** Overview of variables described in Ohio and Michigan, USA (from May 2019 to August 2021) within each 5‐m radius microhabitat plot and how they were used in analysis. For the “coverage” measurements, all nonzero values were multiples of 5. More details are available in the Appendix A.

Variable	Description
Water depth measures	
Water depth (cm)	One measurement in the center of the plot and four at cardinal directions, 5 m from center point. Maximum depth recorded = 100 cm. Mean of 5 measurements used for analysis.
Max. water depth within emerg. veg. (cm)	Water depth within herbaceous emergent vegetation from three pts that appeared the deepest, each separated by at least 1 m. Mean of 3 measurements used for analysis.
Proximity measures	
Distance to nearest large open water (m)	Distance from plot center to nearest large open water patch, defined as any water patch ≥10 m^2^ with no emergent vegetation interspersed. Measured in field and/or using satellite imagery.
Distance to edge of wetland (m)	Distance from plot center to edge of wetland impoundment (e.g., a dike or nonwetland habitat). Measured in the field and/or using satellite imagery.
Distance to woody veg. (m)	Distance to nearest woody vegetation of any size detected. For analysis, noted “yes” or “no” to answer the question, “was woody vegetation ≤50 m from point?”
Plot coverage measures (generally sum to 100)	
% open water	% of plot covered by open water more than 25 cm from emergent vegetation.
% water near veg.	% of plot covered by water within 25 cm of emergent vegetation.
% moist soil/litter	% of plot covered by moist soil, or leaf litter lying (< 25 degree angle relative to ground) on moist ground, within which was interspersed ≥1 vegetation unit per every 5 cm^2^.
% dry soil/litter	% of plot covered by dry soil or leaf litter lying (<25 degree angle relative to ground) on dry ground.
% mud	% of plot that was mud, with <1 vegetation unit per 5 cm^2^.
% herb. emerg. veg. “living”	% of plot covered by standing (≥25 degrees relative to ground) herbaceous emerg. plants, like *Typha* spp., that had any green on them.
% herb. emerg. veg “dead”	% of plot covered by standing (≥25 degrees relative to ground) herbaceous emerg. plants, like *Typha* spp., that had no green on them.
% woody emerg. veg.	% of plot covered by woody emergent plants, like buttonbush (*Cephalanthus occidentalis*).
% upland veg.	% of plot covered by veg. generally found in uplands, not tolerant of prolonged inundation, like northern red oak (*Quercus rubra*).
Vegetative composition, structure, and density measures	
% cover by plant category	% of plot covered by each plant category covering at least 5% of plot. Marsh species were noted and later assigned to categories based on genera. Non‐marsh species were categorized broadly (e.g., “upland tree”). Categories included in analysis if they were “most dominant” (greater percentage than any other plant category) at a plot at least once.
Robel pole	Lowest section on Robel pole mostly visible from 5 m away, viewed at 1.2 m above ground, from cardinal directions. Mean of 4 measures. 0 = 10 cm or less, 1 = 10.01 to 20 cm, etc. Max = 16.
Interspersion	Amount of edge between water/mud/moist soil and vegetation (small, medium, large). See Appendix A for image that shows category criteria.
% emerg. veg. ≥1 m in height	% of standing (≥25 degrees relative to ground) emergent vegetation ≥1 m in height (above water).
% emerg. veg. <1 m in height	% of standing (≥25 degrees relative to ground) emergent vegetation <1 m in height (above water).

### 2.4 Microhabitat analysis

We conducted a Classification and Regression Tree (CART) analysis to describe microhabitat selection. A CART approach is nonparametric and provides an easily interpretable classification tree that categorizes observations based on their characteristics. CART analysis is appropriate for exploratory studies such as ours given that collinearity of explanatory variables is not an issue (Jarošík, 2019). At least 200 observations for binary response variables are recommended for CART analysis (Jarošík, 2019), a criterion that our dataset satisfied (*N* = 607). Our model indicates which variables at what values most effectively categorized points as homing or random. Specifically, the classification tree that we built indicates what conditions within home ranges in our study were associated with points known to be used by king rails and what conditions were associated with points not being used when homing occurred.

We began by excluding all observations that were not used to build home ranges to ensure that inferences pertained to microhabitat. Analysis of autocorrelation plots (via the “acf” function in R v.4.0.3; R Core Team, 2020) for a subset of individuals (*N* = 5) and representative variables (*N* = 4; one from each category in Table 1) indicated independence of our observations given that repeated measures after ≥24 h did not result in autocorrelation between subsequent microhabitat surveys at homing points. Mean Pearson's correlation coefficient, *r*, absolute values from the subset of variables were as follows: distance to open water = 0.20 ± 0.11 (SE), Robel = 0.33 ± 0.08, percent water near vegetation = 0.38 ± 0.1, and water depth = 0.43 ± 0.11.

A strength of CART analysis is that it can handle missing data (Jarošík, 2019). Therefore, we maintained some observations (*N* = 17) that due to observer error or other factors were missing ≥1 variable values if what was missing did not affect other variable values. In addition to those described in Table 1, we included the following variables in the initial CART model: Julian date, year, diel period of day when point was marked (morning, mid‐day, or evening), number of herbaceous emergent species that covered at least 5% of plot, and most dominant plant category. For percent cover by plant category (Table 1) and most dominant plant category, all marsh obligate species were categorized at the genus level with two exceptions: sedges and rushes were combined into a Marsh *Carex*/*Juncus* category and unidentified grass species were combined into Other Marsh Grass due to their structural similarity. We categorized upland species into the following groups: Upland Forb/Graminoid, Upland Shrub/Vine, and Upland Tree (see Appendix A).

We used the rpart package (version 4.1.16; Therneau & Atkinson, 2019) in R v.4.0.3 (R Core Team, 2020) to generate a classification tree. We maintained default values of the rpart.control function except for the cp value, which we changed to 0.005 to ensure that an extensive tree with many splits was built that could then be pruned (Therneau & Atkinson, 2019). We pruned our tree, to improve generality, based on a modification of the description by Jarošík (2019). We identified the ideal number of splits by calculating cross‐validation error rates (xerror) for our model 10 times and then chose the number of splits that was most often within one standard deviation of the minimum xerror.

We used the Wilcoxon test function in R v.4.0.3 (R Core Team, 2020) to compare the number of crayfish caught per trapping session between homing and random points. We also compared the number of crayfish caught per trapping session at points (homing and random treated the same) in the impoundment where king rail home ranges extensively overlapped (see below) to the number of crayfish caught per trapping session at points in the rest of the impoundments where king rails were tracked. We used two‐tailed tests and chose an alpha of 0.05 to indicate statistical significance.

## 3 RESULTS

### 3.1 Trapping

We caught 14 king rails (1 in 2019, 5 in 2020, 8 in 2021). The earliest capture was 14 May and the latest was 15 June. We were able to genetically determine the sex of 13 individuals (4 females, 9 males). While we assumed that the unsexed bird was a male given its larger size (338 g; Perkins et al., 2009), it apparently left the study area before its territory stabilized and thus was removed from the analysis. On two occasions, we caught multiple birds in the same walk‐in trap at the same time. In one instance, a male and female were caught together, and in another case, two males and a female were caught together. We did not re‐capture or observe banded birds between years.

### 3.2 Home range

We determined home‐range size for the 10 birds (*N* = 10; 3 females, 7 males) whose home‐range size stabilized. Mean home‐range size was 8.8 ha (±1.63 [SE]; range = 1.9 to 15.8 ha; Table 2). Home‐range size did not differ between sexes (male 95% CI = 4.46–12.4; female 95% CI = 3.39–15.93 ha) and was the largest for the 2 individuals whose nests were found (Table 2). Mean core‐area size was 1.09 ha (±0.18 [SE]; range = 0.3 to 2.2 ha; Table 2) and did not differ between sexes (male 95% CI = 0.66–1.58 ha; female 95% CI = 0.31–0.95).

**TABLE 2:** Home‐range size estimates, and number of points used to estimate home‐range size, for each king rail (sex and year indicated) whose home range stabilized during our study in Ohio and Michigan, USA (from May 2019 to August 2021).

Bird ID	No. homing Points	KD 95 (ha)	KD 50 (ha)
2020_Male1	31	6.1	1.1
2020_Male2	28	5.8	1.0
2021_Male3	36	15.5	1.2
2021_Male4	17	4.8	0.6
2021_Male5	20	1.9	0.3
2021_Male6	14	9.1	1.5
2021_Male7	15	15.8	2.2
2020_Female1	18	5	0.4
2020_Female2	19	15.8	1.1
2021_Female3	18	8.2	1.5

*Note*: KD 95 = 95% kernel density home range; KD 50 = 50% kernel density core use area. 2020_Female2 successfully hatched chicks and 2021_Male7 presumably did. In both cases, homing did not occur when the radio‐tagged bird was incubating eggs.

**FIGURE 3:**
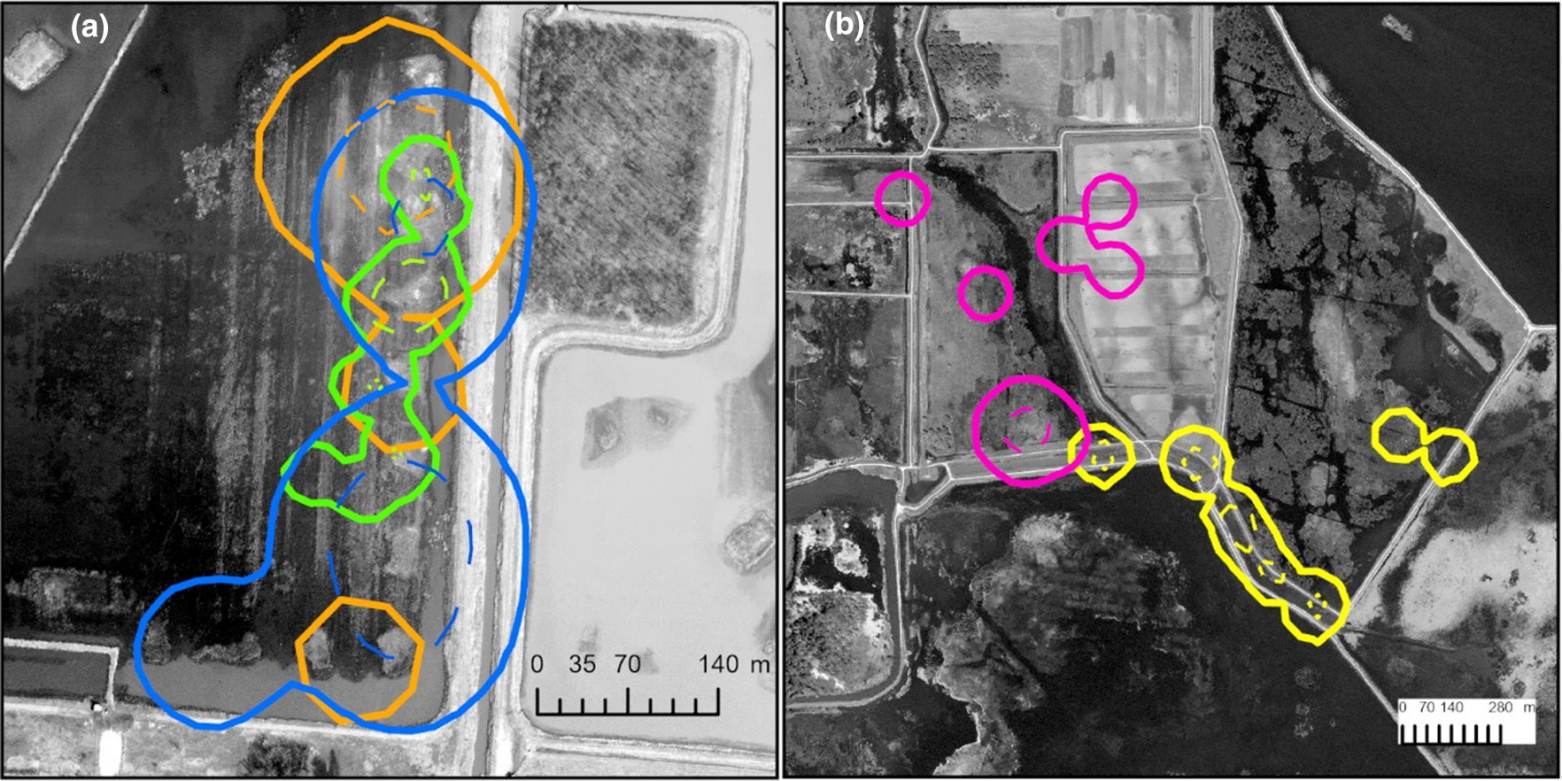
Examples of king rail 95% kernel density (KD) home ranges (solid lines) and 50% KD core areas (dashed lines) during our study in Ohio and Michigan, USA (from May 2019 to August 2021). Note that panel A has a larger scale than panel B. (a) Home ranges of neighboring birds that overlapped extensively. Green = 2021_Male5; orange = 2021_Male4; blue = 2021_Female3. (b) Home ranges of neighboring birds that overlapped very little. Yellow = 2021_Male 7; pink = 2021_Male3.

The degree of home‐range overlap varied between different sets of neighbors, which we defined as individuals with home‐range centroids <800 m apart. For example, home ranges of neighboring male king rails did not substantially overlap in one case (Figure 3). Less than 1% of the area that their home ranges together encompassed included an overlap zone and their core areas did not overlap. In another case, the home ranges of three neighboring king rails (two males and a female) did greatly overlap, whereas their core areas did not (Figure 3). Of these three home ranges, the largest (a female's) encompassed 99% of one male's home range and 75% of the other male's home range, though approximately 50% of its area did not overlap another home range. Of the 5.23 ha that the two male's home ranges together encompassed, 1.27 ha (24.2%) included overlap between them. None of the core areas for these three birds shared more than 10% of their core area with another, though the same 0.01 ha area was used by each bird (Figure 3). A fourth radio‐tagged individual, a male, briefly occurred within the area of home‐range overlap until that individual was depredated. For both of the confirmed‐breeding pairs in our study, only one of the birds in the pair was radio‐tagged. In one impoundment, two birds had home ranges that substantially overlapped between years, with a 1.98‐ha (15.4%) overlap zone of the 12.82‐ha area that their home ranges collectively covered.

Among king rails whose home range stabilized, the mean size of impounded marshes (*N* = 7) where one or more individuals predominantly occurred was 47 ha (range = 2.5–120). All king rail home ranges overlapped at least one impoundment dike. All areas where king rail home ranges stabilized (Figure 1) included contiguous wetland spanning numerous diked impoundments, exceeding 1000 ha in total size. At one of the sites where a king rail home range did not stabilize, the impoundment where the bird (a female) was depredated was <10 ha and the larger contiguous wetland area was <100 ha.

### 3.3 Microhabitat

For the same birds whose home range stabilized (*N* = 10), we analyzed 206 microhabitat surveys conducted at homing points and 401 surveys conducted at random points. The homing points that these surveys describe occurred across three periods: 227 occurred in the morning, 201 occurred during mid‐day, and 179 occurred in the evening. Variable values within homing and random categories tended to be similar between these periods. Our initial model output resulted in year being the least important variable (furthest from the root) included in the pruned tree. We then inspected the variable importance output from the model and found that year was in a three‐way tie for being the tenth most important variable for categorizing points. This indicated that year was relatively unimportant for classification. Though year effects are important to consider, we excluded year from our model to better describe factors that managers could potentially control and then generated another classification tree. We chose the optimal number of splits as described above to create the final, pruned tree that we report.

**FIGURE 4:**
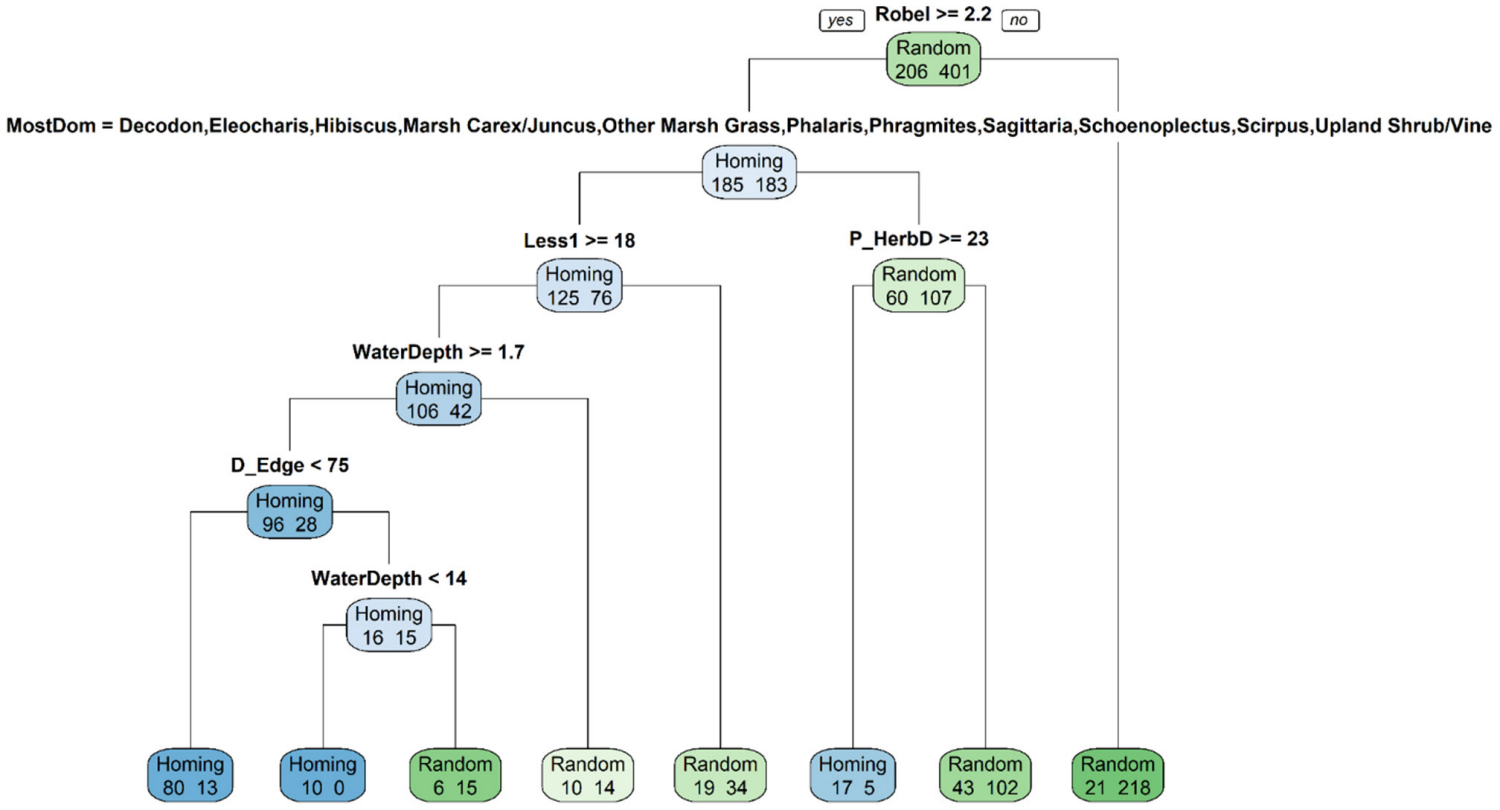
Results from CART analysis for our study in Ohio and Michigan, USA (from May 2019 to August 2021). To interpret the classification tree, start at the root (top) and note that an answer of “yes” results in going left and categorizing points as “homing” (king rail present) whereas an answer of “no” results in going right and typically categorizing points as “random” (king rail absent). Within each node, except for the root node, the larger number represents how many points therein were accurately categorized as the specified category based on the previous question and the smaller number indicates how many were inaccurately categorized. Each path to the terminal nodes represents a different combination of conditions associated with King Rail presence or absence. The Robel pole (vegetative density) measurement was the best indicator of whether or not points were used by a king rail. All values pertain to measurements within a 5‐m radius plot. MostDom = Most dominant plant species; Less1 = Percent of emergent vegetation less than 1 m in height; P_HerbD = Percent coverage by dead (not green) herbaceous emergent vegetation; WaterDepth = Mean water depth (cm); D_Edge = Distance (m) to edge of wetland.

Our classification tree achieved ~81% accuracy (Figure 4), with 490 of 607 points accurately identified as homing (king rail present) or random (king rail absent). Approximately 52% of homing points (107 of 206) and 96% of random points (383 of 401) were correctly identified. The classification tree (Figure 4) indicated that the Robel pole measurement, which quantifies vegetative density (Table 1), was the most important variable for categorizing points as homing or random. Specifically, using a Robel pole measurement of <2.2 to indicate a random point resulted in ~36% of all (*N* = 607) survey points being correctly classified (*N* = 218) as a random point and only ~3% of homing points being incorrectly classified as a random point (*N* = 21). For points with a Robel pole measurement ≥2.2, the most dominant plant species was the next splitting variable to consider during the classification process (Figure 4). A variety of marsh plant groups (11), including those in the genera *Carex*, *Juncus*, *Phalaris*, and *Phragmites*, were associated with king rail presence whereas the genus *Typha* was not associated with king rail presence. Further down the classification tree, percentage of emergent vegetation that was short (<1 m) and coverage by senesced herbaceous emergent vegetation was positively associated with king rail presence. Additionally, water depths of ≥1.7 cm and <14 cm were positively associated with king rail presence, as was being <75 m from the marsh's edge (Figure 4).

Examination of CART output indicated that several variables on their own were as effective, or more effective, at categorizing points than those ultimately included in our classification tree. However, these variables were not included at any node because another variable was, at each node, better at categorizing remaining points as homing and random. These important but unused variables were: percentage of emergent vegetation ≥1 m in height (>12.5% associated with homing), percentage of plot covered by living herbaceous emergent vegetation (>12.5% associated with homing), percentage of plot covered by open water (<7.5% associated with homing), and maximum water depth within herbaceous emergent vegetation (<21.5 cm associated with homing). Strong correlations between predictor variables were rare, with only three pairs of variables having Pearson's correlation coefficient |r| values >0.6: percentage upland forb/graminoid and percentage upland = 0.85; water depth and percent open water = 0.69; percent short (<1 m) vegetation and percent tall (≥1 m) vegetation = 0.61. We did not remove any of these variables during analysis because of their potential use as surrogate variables.

An overview of quantitative variables included in the CART analysis, and how they compared between homing and random points, is provided (Table 3). Note that these parameter estimates can be used to evaluate variables in isolation regarding king rail presence and should supplement inferences drawn from our classification tree (Figure 4), which includes values for variables as they relate to king rail presence given defined environmental conditions. Variables from all four of the broad categories overviewed in Table 1, except for proximity measures, differed between homing and random points. Note that some variables, like percent open water, were clearly distinct between homing and random points but were not included in our classification tree (Figure 4).

**TABLE 3:** Mean, 95% confidence interval (CI), and interquartile range (IQR) for nearly all quantitative variables included in the CART analysis for our study in Ohio and Michigan, USA (from May 2019 to August 2021).

Variable	Homing (*N* = 206^a^)			Random (*R* = 401^b^)		
	Mean and CI		IQR	Mean and CI		IQR
**Percent herbaceous emerg. veg. living**	**40.9**	**38.3–43.5**	**30–50**	25.9	23.4–28.4	1–44
**Percent herbaceous emerg. dead (P_HerbD)**	**12.6**	**11.2–14.1**	**5–20**	7.4	6.5–8.3	0–10
Percent woody emerg. veg.	0.3	0.0–0.6	0–0	0.2	0.1–0.3	0–0
Percent upland veg.	6.5	4.8–8.3	0–5	9.9	7.8–12.1	0–5
Percent dry soil/litter	4.7	3.5–6.0	0–5	6.1	4.9–7.2	0–5
Percent moist soil/litter	7.1	5.7–8.4	0–10	5.1	4.2–6.0	0–5
Percent mud	1.5	1–2.1	0–0	1.2	0.6–1.8	0–0
**Percent open water**	1.4	0.8–2.0	0–0	**22.8**	**19.1–26.5**	**0–30**
Percent water near veg.	25	22.6–27.4	10–39	20.9	18.6–23.2	0–35
Percent *Butomus*	2.3	1.4–3.3	0–0	3.4	2.3–4.4	0–0
**Percent *Hibiscus***	**3.6**	**2.1–5.0**	**0–0**	1.5	1.0–2.0	0–0
**Percent Marsh *Carex/Juncus***	**6.4**	**4.8–8.0**	**0–10**	2.5	1.8–3.3	0–0
Percent *Persicaria*	1.6	0.6–2.6	0–0	2.9	1.9–3.9	0–0
**Percent *Phalaris***	**8.7**	**6.4–10.9**	**0–10**	4.7	3.4–6.0	0–0
**Percent *Phragmites***	**8.5**	**6.5–10.5**	**0–15**	4.1	3.1–5.2	0–0
**Percent *Scirpus***	**2.3**	**1.0–2.5**	**0–0**	0	0–0	0–0
Percent *Typha*	13	10.2–15.8	0–20	9.8	8.1–11.4	0–15
**Percent upland forb/graminoid**	1.7	0.8–2.6	0–0	**6.3**	**4.5–8.2**	**0–0**
Percent upland shrub/vine	3.3	2.2–4.3	0–0	2.4	1.6–3.3	0–0
Percent upland tree	0.7	0.3–1.0	0–0	0.5	0.3–0.7	0–0
Percent herbaceous emerg. veg. < 1 m in height (Less1)	38.1	34.2–42.1	15–60	42.9	39.2–46.5	5–75
**Percent herbaceous emerg. veg. >1 m in height**	**61.4**	**57.4–65.3**	**40–85**	42	38.4–45.6	0–80
**Number of herbaceous emerg. plant spp.**	**2.7**	**2.5–2.8**	**2–3**	1.7	1.6–1.8	0–3
**Mean water depth (cm) (WaterDepth)**	13.3	11.9–14.7	6.3–17.5	**27.3**	**24.7–29.9**	**6.3–39.2**
**Mean max water depth in emerg. veg. (cm)**	15.2	13.8–16.6	8.3–20.0	**20.6**	**18.7–22.5**	**2.3–32.0**
**Mean Robel (Robel)**	**7.2**	**6.6–7.7**	**4.5–10**	3.5	3.1–4.0	0–6
Distance to open water (m)	44.7	39.5–50.0	15–66.8	38.8	34.1–43.6	5–54
Distance to edge (m) (D_Edge)	70.3	62.3–78.3	35–86.0	72.4	66.7–78.7	17.4–109.9

*Note*: The only variables excluded are some genera categories for plants that had a mean coverage of <2% for both homing and random points. Codes in parentheses occur in Figure 4. Variable names are bolded if the confidence intervals for the homing and random points do not overlap. Mean, confidence interval, and interquartile range are bolded for the point type that was greater.

^a^ Except “percent emerg. veg. more” (AND less) “than 1 m in height” (both *N* = 199).

^b^ Except “Robel” (*N* = 400) and “percent emerg. veg. more” (AND less) “than 1 m in height” (both *N* = 396).

*Typha* spp. was most often the most dominant plant category at both homing and random points (Table 4). *Typha* spp. was also, among all plant categories, most commonly present at random points, whereas *Phragmites* was most commonly present at homing points (Table 4). Marsh *Carex*/*Juncus* spp. and *Scirpus* spp., among others, were more common as the most dominant plant category and were more commonly present at homing points than at random points (Table 4). Two categorical variables included in CART analysis were not included in Table 4. Distance to woody vegetation did not vary between homing and random points, with 56% (*N* = 115) of homing points within 50 m of woody vegetation and 53% (*N* = 211) of random points within 50 m of woody vegetation. Compared to random points, homing points were more likely to have large interspersion (57% vs. 27%), as likely to have medium interspersion (both 24%), and less likely to have small interspersion (10% vs. 25%).

**TABLE 4:** All plant categories that were included in CART analysis, proportion/number of points where those categories were most dominant, and proportion/number of points where those categories were present at homing and random points for our study in Ohio and Michigan, USA (from May 2019 to August 2021).

Plant category	Homing (*N* = 206)		Random (*N* = 401)	
	Most Dominant	Present	Most Dominant	Present
*Typha*	0.25 (51); [8]	0.43 (89); [10]	0.23 (93); [10]	0.41 (166); [10]
*Phragmites* ^a^	0.17 (36); [8]	0.46 (92); [10]	0.09 (37); [9]	0.24 (95); [10]
**Marsh *Carex/Juncus*** ^a^	**0.16 (32)**; [6]	**0.32 (65)**; [9]	0.04 (18); [4]	0.14 (56); [10]
*Phalaris* ^a^	0.15 (30); [8]	0.38 (79); [10]	0.09 (36); [10]	0.21 (86); [10]
***Scirpus*** ^a^	**0.05 (10); [2**]	**0.08 (16); [4]**	0 (0); [0]	0 (1); [1]
Upland Shrub/Vine^a^	0.05 (10); [4]	0.21 (43); [6]	0.03 (13); [5]	0.15 (60); [8]
**Upland Forb/Graminoid**	0.04 (8); [5]	0.11 (22); [6]	**0.09 (37); [10]**	0.17 (69); [10]
**Multiple**	0.04 (8); [7]	N/A	**0.09 (35);** [10]	N/A
*Hibiscus* ^a^	0.03 (6); [1]	0.18 (37); [7]	0 (1); [1]	0.14 (58); [9]
***Butomus***	0.01 (3); [3]	0.17 (34); [7]	**0.06 (26); [6]**	0.16 (66); [7]
*Decodon* ^a^	0.01 (3); [1]	0.03 (7); [1]	0.01 (3); [1]	0.02 (8); [2]
***Persicaria***	0.01 (3); [2]	0.09 (18); [8]	**0.05 (20); [7]**	0.13 (52); [8]
***Eleocharis*** ^a^	0.01 (2); [1]	**0.06 (13);** [3]	0 (2); [1]	0.03 (12); [5]
Other Marsh Grass^a^	0.01 (2); [1]	0.02 (4); [2]	0 (0); [0]	0 (0); [0]
*Sagittaria* ^a^	0 (1); [1]	0.01 (3); [3]	0 (0); [0]	0 (1); [1]
*Schoenoplectus* ^a^	0 (1); [1]	0.04 (9); [4]	0 (0); [1]	0.02 (8); [3]
**None**	0 (0); [0]	N/A	**0.17 (69); [9]**	N/A
*Sparganium*	0 (0); [0]	0.01 (3); [2]	0.01 (6); [2]	0.03 (14); [4]
Upland Tree	0 (0); [0]	0.10 (20); [4]	0.01 (3); [3]	0.06 (25); [6]
***Asclepias***	0 (0); [0]	**0.12 (24);** [4]	0 (1); [1]	0.05 (20); [8]
***Lythrum***	0 (0); [0]	**0.12 (24);** [7]	0 (1); [1]	0.04 (15); [7]

*Note*: Counts for each cell are in parentheses. Numbers in brackets indicate how many of the 10 King Rails had the specified plant category as most dominant and/or present at one or more points. Plant category names are bolded if that category has a proportion value in the “most dominant” or “present” columns of at least 0.05 that is at least twice as large for one of the point types (homing or random) compared to the other. Values are bolded within the point type columns for each value that is at least twice as large as the other point type's value for that category. Multiple indicates that multiple plant categories were tied for most dominant. “None” indicates that no species covered ≥5% of the plot.

^a^ When the most dominant species, our classification tree associated this plant category with king rail presence.

We did not include crayfish abundance in the CART analysis due to relatively low sample size (*N* = 38). Contrary to our prediction, crayfish abundance per trapping session was not greater (*W* = 637, *p* = 0.579) at homing points (mean = 0.9; *N* = 38) compared to random points (mean = 1.3; *N* = 38). Crayfish abundance was, however, greater (*W* = 287, *p* < .001) within the wetland impoundment (Figure 3) where three king rail home ranges overlapped (mean = 3.78; *N* = 14) than in the other impoundments (mean = 1.25; *N* = 24). The Hodges‐Lehmann estimator indicated three more crayfish (95% confidence interval = 1 to 4) at homing and random points in the impoundment with extensive home‐range overlap compared to the other impoundments.

**FIGURE 5:**
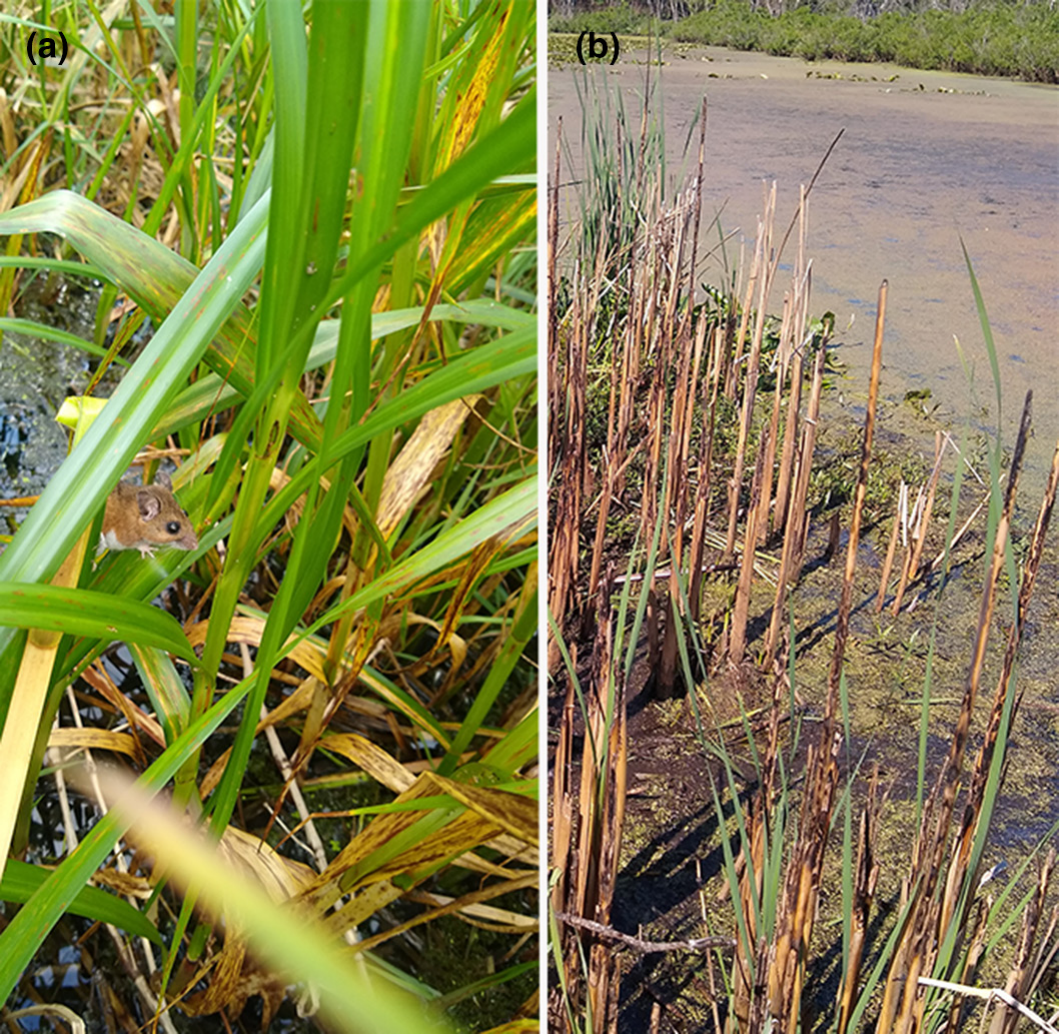
Example of a homing location (a), where a king rail occurred, and a random location (b), where we did not document a king rail occurring. Note how at the homing location dense herbaceous vegetation (*Scirpus fluviatilis*, in this case) is emerging from shallow water (<15 cm). Conversely, at the random location sparse vegetation (*Typha* sp., primarily) is occurring beside deep (>25 cm), open water.

Overall, our microhabitat analyses indicate that king rails tended to be associated with dense (Figure 4, Table 3), robust vegetation consisting of herbaceous plants such as those in the categories *Scirpus* and Marsh *Carex/Juncus* (Tables 3, 4). Shallow water, usually between 6 and 17 cm (Table 3) but as little as 1.7 cm in some cases (Figure 4), was also associated with king rail presence. We provide a picture of a typical point where king rails tended to occur and another of a typical point where king rails tended not to be documented (Figure 5).

## 4 DISCUSSION

Our results support our hypothesis that king rails in western Lake Erie coastal wetlands use microhabitat nonrandomly and these results could help conservationists identify and restore or manage suitable conditions for this species. We also provide an estimate of king rail home‐range size and describe different degrees of home‐range overlap which could help to inform population estimates. Note that though our sample size was small (10 king rails) and should, therefore, be interpreted with caution especially given proximity of focal birds, the estimated number of king rails that occur in the entire UMGL region is <400 (Cooper, 2008). Thus, our study is likely highly applicable to western Lake Erie coastal wetlands and may also be relevant to other parts of the Great Lakes region where similar wetlands occur.

The mean breeding season home‐range size of king rails in our study (8.8 ha) is smaller than was found in a study in North Carolina (22.5 ha, for 13 individuals; Kolts & McRae, 2017), though their 95% confidence interval (9.98–36.02 ha) overlapped with ours (5.61–11.99 ha). Kolts and McRae (2017) found king rails also had a larger mean core‐area size (3.1 ha) than occurred in our study (1.1 ha), though again their 95% confidence interval (0.94–5.26 ha) overlapped with ours (0.74–1.44 ha). King rails in our study had a larger mean home‐range size than Pickens and King (2013) found at their primary site in Texas, where mean home‐range size for 18 individuals was 4.4 ha (95% CI = 3.22–5.58). They described a mean core‐area size of 0.9 ha which was very similar to our findings. Factors such as conspecific density, food availability, and habitat structure could have influenced home‐range size variability between individuals within our study (Table 2). These factors, as well as different approaches for choosing the kernel density smoothing parameter (Seaman & Powell, 1996), could have resulted in some of the variability in home‐range and core‐area size estimates in our study compared to the other studies discussed. Both Pickens and King (2013) and Kolts and McRae (2017) studied king rails in marine coastal wetlands where phenomena, like storm surges, occur that are generally absent in the Great Lakes region and so could lead to different adaptations.

Pickens and King (2013) reported “minimal” overlap of king rail home ranges. This is contrary to what we found in our study wherein three adult king rails each had extensive (≥50%) home‐range overlap with at least one other home range while displaying relatively little (<10%) core‐area overlap (Figure 3). Kolts and McRae (2017) reported home‐range overlap soon after chicks had hatched though did not quantify degree of overlap. The relatively high within‐impoundment density of crayfish, a known king rail food source (Pickens & Meanley, 2020), may have been a primary cause for the instance of extensive home‐range overlap in our study given that no nesting behavior was detected. The female's larger home range may have been a consequence of mate prospecting (Kesler & Haig, 2007). It is possible that extensive home‐range overlap and little core‐area overlap for nonmated birds is commonly displayed by king rails in western Lake Erie coastal wetlands in prey‐rich areas, though more study is required to explore that possibility. Such space use, if common, may indicate a degree of flexibility regarding spatial organization that could be useful knowledge for managers. We recommend that estimates of king rail population size within a given area in western Lake Erie coastal wetlands assume that individuals require at least the largest, nonoverlapping home‐range area that we report (15.8 ha) to avoid overestimation.

We found that vegetative density (for multiple plant categories, heights, and stages), most dominant plant species, and water depth were particularly important for predicting third order microhabitat use by king rails (Figure 4). In our study, 75% of the points where king rails occurred were densely vegetated such that, when viewed from 5 m away, the lowest point clearly visible was on average between 45 and 110 cm above the water surface (or ground if no water; Table 3). We presume that this degree of vegetative coverage, which has been associated with king rails previously (Pickens & King, 2014), provides sufficient cover to avoid ground‐based and aerial predators (Pickens & Meanley, 2020). It is possible, however, that our method of homing caused individuals to seek denser vegetation than they generally use. Similar to Pickens and King (2013), we found that *Phragmites* (mostly invasive in our study area and not in theirs) and *Schoenoplectus* spp. were associated with king rail presence. However, we did not find that *Typha* spp. were associated with king rail presence, whereas Pickens and King (2013) did. The majority of *Typha* spp. present in our study area were either the hybrid variety, *Typha* x *glauca*, or the exotic species, *Typha angustifolia*, rather than the native, *Typha latifolia*. Given the known negative effects of invasive *Typha* spp. on other plant and animal spp. (Frieswyk & Zedler, 2007; Lishawa et al., 2020), it is possible that Pickens and King (2013) had less invasive *Typha* spp. in their study area than we did which resulted in more use of that plant category by king rails. The association between king rails and *Phragmites* that we and Pickens and King (2013) found could be due to the preference of king rails for relatively shallow water depths, where *Phragmites* tends to grow. Other plant categories that we found king rails commonly associated with, like Marsh *Carex*/*Juncus* spp. (Table 4), also co‐occurred with shallow water and could represent the vegetative community that king rails primarily utilized before invasive *Phragmites* spread across the lower Great Lakes region in the late 1990s (Wilcox, 2012). The association with *Phragmites* by king rails in our study may contrast with studies that have found that *Phragmites* negatively impacts other marsh bird species (Meyer et al., 2010; Robichaud & Rooney, 2017). However, *Phragmites* within much of our study area had been actively managed for approximately 10 years and so patch size and density may have been less than what was observed by previous investigators (Meyer et al., 2010, Robichaud & Rooney, 2017). We speculate that the negative impacts of *Phragmites* might not be observed until the landscape is dominated by large monocultures (Benoit & Askins, 1999). Similar to Pickens and King (2013), we found that king rails were associated with greater emergent herbaceous plant species richness (Table 3), which generally is not associated with *Phragmites* presence in the Great Lakes region (e.g., Bonello & Judd, 2019). Microhabitat appropriate for king rails likely shifts in bands within nonimpounded, coastal wetlands as Great Lakes water levels fluctuate and could be reduced in extent by expansion of non‐native *Typha* spp. following a reduction in water levels (Frieswyk & Zedler, 2007).

King rails generally occurred (75% of points; Table 3) where water depth was ≥~6 cm and ≤~17 cm, which presumably sustains favorable vegetative conditions and food availability during the breeding season. Note that our classification tree (Figure 4) indicates water depths associated with king rail presence in specific rather than general conditions given that they occur at or near terminal nodes. The water depth range that we observed individuals using in our study could also deter predators from accessing king rails (Picman et al., 1993). Pickens and King (2013) found that king rails often occurred where water depth was zero, though they indicated that this may have been due to an unusually dry study period. Given that many wetlands in our study area are impounded, managers often have some control over water depth conditions. However, impoundments managed for waterfowl are often completely de‐watered in the spring or summer to facilitate moist‐soil plant germination or kept at water depths of >17 cm to facilitate emergent vegetation establishment. We recognize that impoundments cannot be solely managed to benefit king rails, but impoundments with sufficient variation in elevation could be managed to provide a mix of shallow water habitat that is useful for king rails and deeper water habitat that provides value to other wetland‐dependent wildlife and to waterfowl hunters. Wading birds, shorebirds, and some dabbling ducks can also benefit from shallower (<20 cm) water depths (Colwell & Taft, 2000; Pickens & King, 2014) and so could benefit from impoundments managed to facilitate king rail presence. If managers successfully promote king rail presence, home ranges in western Lake Erie coastal marshes likely will occur near wetland edges (e.g., Figure 3) and so king rails could be affected by disturbance such as mowing and vehicle/foot traffic that often occurs on dikes of impounded wetlands. Meso‐predators could also frequently travel upon dikes and so pose a threat to king rails (Frey & Conover, 2006), which warrants investigation. King rail conservation efforts would also be advanced by determining the generality of our results via similar investigations in other sections of the Great Lakes region.

Some similarities exist between our microhabitat results and the findings of investigators who made inferences at a broader geographical scale. For example, we found that king rails tended not to be associated with woody vegetation within home ranges, similar to what Bolenbaugh et al. (2012) qualitatively suggested based on habitat descriptions at sites where king rails were detected within the UMGL Joint Venture boundaries. Darrah and Krementz (2009) also found, in one of the years of their study near rivers in Missouri and Illinois, that the probability of king rail occupancy declined with increasing coverage of woody vegetation. However, Glisson et al. (2015) found a positive association of king rail occupancy with scrub‐shrub wetlands. Darrah and Krementz (2009) reported, similar to our findings, a positive relationship between king rail presence and the degree of interspersion. Kane (2020), who studied king rails in western Lake Erie coastal wetlands, also found a positive association with interspersion. Contrary to the findings of Darrah and Krementz (2009), we found evidence that tall emergent vegetation (≥1 m) was associated with king rail use overall (Table 3) and that, when particular conditions were met (Figure 4), so was short (<1 m) emergent vegetation. Darrah and Krementz (2009) noted that *Typha* spp. was the most prominent tall (>1 m) emergent plant category present at sites where king rails occurred. Bolenbaugh et al. (2012) also noted *Typha* spp.—as well as a variety of other plant categories—at sites where king rail occurred, though predicted that invasive *Typha* spp. are negatively associated with king rail presence. Though king rails did not preferentially select *Typha* spp. within home ranges in our study, that plant category was ubiquitous (Table 4) and used in proportion to its availability. At broader scales of habitat selection (i.e., when home ranges are being selected), it may also be used in proportion to its availability or even preferred relative to other plant categories. We recommend further study to experimentally determine if controlling invasive species, such as *Typha* and *Phragmites* spp., increases king rail patch use and/or reproductive success.

In summary, our results suggest that managers of western Lake Erie coastal marshes should aim to provide ≥~16 ha of suitable, contiguous habitat to support a king rail home range. Vegetation should be dense enough to completely obscure a king rail from sight when viewed from 5 m away. When possible, managers should attempt to provide water depths of 6 to 17 cm between May and August, during the breeding season. King rails with chicks may prefer a water depth of less than 1 cm (Darrah & Krementz, 2011) which underscores the necessity of providing a variety of shallow water depths to suit multiple needs. The presence of *Phragmites* emerging from shallow (<20 cm) water could indicate that a wetland patch is hydrologically appropriate for king rails. Though our study indicates that king rails frequently use *Phragmites* stands in the Great Lakes region, dense monocultures are known to be detrimental to marsh‐nesting birds (Meyer et al., 2010). We, therefore, recommend promoting a diverse mix of native, robust herbaceous emergent vegetation—such as certain wetland obligate species in the genera *Carex* and/or *Juncus*—to increase the likelihood of use by king rails.

## AUTHOR CONTRIBUTIONS

**Dustin E. Brewer:** Conceptualization (equal); data curation (lead); formal analysis (lead); funding acquisition (supporting); investigation (lead); methodology (lead); project administration (supporting); resources (supporting); supervision (supporting); visualization (lead); writing – original draft (lead); writing – review and editing (equal). **Thomas M. Gehring:** Conceptualization (equal); funding acquisition (equal); project administration (equal); resources (equal); supervision (equal); writing – review and editing (equal). **Madeline M. Garcia:** Formal analysis (supporting); investigation (supporting); writing – review and editing (supporting). **Brendan T. Shirkey:** Conceptualization (equal); investigation (equal); project administration (equal); supervision (equal); writing – review and editing (equal). **John W. Simpson:** Conceptualization (equal); project administration (equal); resources (equal); writing – review and editing (equal). **Auriel M. V. Fournier:** Methodology (supporting); resources (supporting); writing – review and editing (equal).

## CONFLICT OF INTEREST STATEMENT

We had no competing interests.

## Data Availability

Data and code associated with our study are available at this link: https://doi.org/10.5281/zenodo.6604660.

## APPENDIX A

### Microhabitat field protocol

This section describes in detail how microhabitat data were collected, and could be collected to replicate this study, in the field.

#### Plot coverage measures^*,#^

*% Herbaceous emergent vegetation*: Estimate the percentage of the plot that is covered by herbaceous emergent vegetation (e.g., *Typha*, *Phragmites*) which is normally found where inundation occurs for part of the year. Break this down into “living” (green vegetation) and “dead” (nongreen, senesced vegetation). Only include vegetation at or more than 25 degrees relative to ground/water that is at least 15 cm above the water (or ground if no water).

*% Woody emergent vegetation*: Estimate percentage of the plot that is covered by woody emergent vegetation, like buttonbush, which is normally found where inundation occurs for part of year. This includes willows or other tree species that grow in water. Only include vegetation at or more than 25 degrees relative to ground/water that is at least 15 cm above the water (or ground if no water).

*% Upland vegetation*: Estimate percentage of plot that is covered by vegetation that is rarely, if ever, inundated by water. For example, grass or trees on a dike normally found where inundation does not occur (like orchard grass). Only include vegetation at or more than 25 degrees relative to ground/water that is at least 15 cm above the water (or ground if no water).

*% Dry soil/litter*: Estimate percentage of plot covered by dry soil (not squishy) or leaf litter that is lying (at <25 degree angle) on the dry ground or on dry vegetation. This includes muskrat mounds if they are dry. Note that some pieces of litter could be both moist and dry litter at different locations and should contribute to both “dry soil/litter” and “moist soil/litter” categories. Check under litter to determine if it is touching dry or moist substrate. If dry and suspended in air, then litter is in the dry soil/litter category.

*% Moist soil/litter*: Estimate the percentage of the plot covered by soil, not covered by water, that is wet to the touch (squishy when foot on it) and on which is interspersed at least one vegetation unit (including leaf litter) per every 5 cm^2^. This includes floating mats and muskrat mounds if the latter are moist, as well as leaf litter that is lying (at <25 degree angle) on the moist ground or on moist vegetation. Note that some pieces of vegetation could be both moist and dry litter at different locations and should contribute to both “dry soil/litter” and “moist soil/litter” categories. Check under litter to determine if it is touching dry or moist substrate.

*% Mud*: Estimate the percentage of the plot that is mud. Unlike moist soil, mud has fewer than one vegetation unit (including leaf litter) per every 5 square cm.

*% Open water*: Estimate the percentage of the plot that is open water. To be “open water,” there should be no emergent vegetation within 25 cm of any area considered open water, but there can be floating plants like lilies or bladderwort.

*% Water near vegetation*: Estimate percentage of plot that is covered by water that is within 25 cm of emergent vegetation.

*For percent coverage, look straight down from a height of 1.2 m (imagine everything above that height is not there) and estimate the percentage of each cover type that you see in the 5 m radius circle. Percentages should range from 0 to 100. Nonzero values generally should be multiples of 5. If there is just a single specimen for a particular cover type, that is, far less than 5%, record “1%” and subtract 1% from the cover type that covers the most area. If subtraction needs to occur and there are multiple cover types that are tied for most areal coverage, then split the value that needs to be subtracted and subtract that from each cover type that was tied for most coverage. If a human‐created cover (like a paved road) is encountered, only fill in percentages for listed cover types (OK if does not add up to 100).

^#^Species categorizations (herbaceous emergent, woody emergent, upland) are based on where that species generally grows, regardless of where it is growing. *Phragmites* on the side of a dike, for example, would be considered emergent because it generally emerges from water at some point during year. Here are categorizations for commonly occurring plant taxa:

*Herbaceous emergent*: *Typha*, *Phragmites*, Reed Canary Grass (*Phalaris arundinacea*), Swamp Rose Mallow (*Hibiscus moscheutos*), Smartweeds (*Persicaria*), American Bur‐reed (*Sparganium americanum*), Flowering Rush (*Butomus umbellatus*), Broadleaf Arrowhead (*Sagittaria latifolia*), Pickerelweed (*Pontederia cordata*), Purple Loosestrife (*Lythrum salicaria*), Swamp Loosestrife (*Decodon verticillatus*), Cursed Crowfeet (*Ranunculus sceleratus*), *Eleocharis*, River Bulrush (*Scirpus fluviatilis*), Soft‐stemmed Bulrush (*Schoenoplectus tabernaemontani*), *Carex* (growing out of water, amongst emergent species), Swamp Milkweed (*Asclepias incarnata*)
*Upland*: Dike Grass (e.g., Kentucky Blue Grass [*Poa pratensis*]), *Cornus*, Poison Hemlock (*Conium maculatum*), Canada Thistle (*Cirsium arvense*), Eastern Cottonwood (*Populus deltoides*), dry sedges (*Carex* growing out of dry soil, not amongst emergent veg.), common milkweed (*Aclepias syriaca*), Jewelweed (*Impatiens capensis*), Hemp Dogbane (*Apocynum cannabinum*), Creeping Jenny (*Lysimachia nummularia*)
*Woody emergent*: Buttonbush (*Cephalanthus occidentalis*), *Salix*, Swamp Rose (*Rosa palustris*)

#### Water depth measures

*Water depth*: 5 measurements (use meter stick), in center of plot and in 4 cardinal directions 5 m from center point. Max depth is 1 meter (i.e., if deeper than 1 meter, write 1 meter). Record to nearest centimeter. *Take these measurements the day of homing*.

*Max water depth within emergent plants*: Record depths of 3 points that appear the deepest within emergent vegetation. Each point should be at least 1 m apart and within the plot. Record to nearest centimeter. *Take these measurements the day of homing*.

#### Proximity measures

*Distance to large open water patch*: Estimate distance from point to nearest large patch of open water (defined as any water patch where emergent vegetation does not grow that covers at least 10 square meters). Cap this distance at 100 meters (i.e., if there is not any “large” open water within 100 meters, record 100 meters). Value could be 0 if point is in a large open water patch. Record to nearest meter.

*Distance to edge of wetland*: Estimate distance from point to the edge of wetland. Consider “edge” to be top of dike nearest to wetland or the very beginning of nonwetland habitat if not in an impounded wetland.

*Distance to woody vegetation*: Estimate the distance to nearest woody vegetation (e.g., buttonbush, willow). Cap at 100 meters (i.e., if there is not any woody vegetation within 100 meters, record 100 meters). Record to nearest meter.

#### Vegetative composition, structure, and density measures

*% cover by plant species*: Estimate % coverage by all plant species (herbaceous emergent veg, woody emergent veg, and upland veg species) that cover at least 5% of the area covered by vegetation. Total should sum to ≤ total of the three broad categories. Record percentages as described above. Species should later be combined into specific categories, as described below.

*Robel pole*: After placing the Robel pole in the center of the plot, from 5 m away determine the lowest section on the pole that is mostly visible from a height of 1.2 m above your feet. Do this from all cardinal directions.

*% of standing emergent veg. ≥ than 1 meter in height*: Height is above water (not to roots). Estimate percentage of emergent vegetation (woody and herbaceous) greater than or equal to 1 meter in height which is standing (≥25 degrees). % from 0 to 100, with nonzero values as multiples of 5. With the below measure, should sum to 100, or 0 if there was no emergent veg.

*% of standing emergent veg. < than 1 meter in height*: Height is above water (not to roots). Estimate percentage of emergent vegetation (woody and herbaceous) less than 1 meter in height which is standing (≥ 25 degrees). % from 0 to 100, with nonzero values as multiples of 5. With the above measure, should sum to 100, or 0 if there was no emergent veg.

*Interspersion*: Amount of edge between water (any depth)/mud/moist soil and vegetation. Choose from “none,” “small,” “medium,” or “large” using the below figure as a guide. Diamonds are vegetation.

### Species included in plant categories

This section describes how plant species were categorized for CART analysis. Only plant categories that were most dominant at least once are included.

Marsh *Carex*/*Juncus*: Woolly fruited Sedge (*Carex lasiocarpa*), Fox Sedge (*Carex vulpinoidea*), Foxtail Sedge (*Carex alopecoidea*), Wooly Sedge (*Carex pellita*), Crested Sedge (*Carex cristatella*), Soft Rush (i.e., soft‐stemmed rush; *Juncus effusus*), Bristle‐stalked Sedge (*Carex leptalea*), Torey's Rush (*Juncus toreyi*), Bottle brush sedge (*Carex hystericina*), Hop Sedge (*Carex lupulina*), others in *Carex/Juncus* not identified to species.

*Eleocharis*: unidentified species in the genus.

*Scirpus*: Dark‐green Bulrush (Scirpus atrovirens), River Bulrush (*Scirpus fluviatilis*), Wool Grass (*Scirpus cyperinus*).

*Schoenoplectus*: Soft‐stemmed bulrush (*Schoenoplectus tabernaemontani*).

*Typha*: Narrow‐leaved Cattail (*Typha angustifolia*), Broadleaf Cattail (*Typha latifolia*), Hybrid cattail (*Typha x glauca*).

*Asclepias*: Swamp Milkweed (*Asclepias incarnata*), other unidentified marsh species in genus.

*Phragmites*: Common Reed (*Phragmites australis*).

*Persicaria*: Nodding Smartweed (*Persicaria lapathifolia*), Water Smartweed (*Persicaria amphibia*), Mild Water Pepper (*Persicaria hydropiperoides*), other unidentified marsh species in genus.

Upland Forb/Graminoid: Smooth Brome (*Bromus inermis*), Common Milkweed (*Asclepias syriaca*), Kentucky Blue Grass (*Poa pratensis*), Orchard Grass (*Dactylis glomerata*), Poison Hemlock (*Conium maculatum*), Red Clover (*Trifolium pratense*), Sweet Clover (*Meliotus alba*), Virginia Knotweed (*Persicaria virginiana*), Wild Carrot (*Daucus carota*), Canada Thistle (*Cirsium arvense*), Blue Vervain (*Verbena hastata*), Meadow anemone (*Anemone canadensis*), Hemp Dogbane (*Apocynum cannabinum*), Toad Rush (*Juncus bufonius*).

Upland Shrub/Vine: *Cornus*, Autumn olive (*Elaeagnus umbellata*), Virginia Creeper (*Parthenocissus quinquefolia*), *Vitis*, Multiflora rose (*Rosa multiflora*).

Upland Tree: Eastern Cottonwood (*Populus deltoides*), *Fraxinus*, Swamp White Oak (*Quercus bicolor*), Honey Locust (*Gleditsia triacanthos*).

*Butomus*: Flowering Rush (*Butomus umbellatus*).

*Decodon*: Swamp Loosestrife (*Decodon verticillatus*).

*Hibiscus*: Swamp Rose Mallow (*Hibiscus moscheutos*).

*Lythrum*: Purple Loosestrife (*Lythrum salicaria*).

*Phalaris*: Reed Canary Grass (*Phalaris arundinacea*).

*Sagittaria*: Broadleaf Arrowhead (*Sagittaria latifolia*), other *Sagittaria* not identified to spp.

*Sparganium*: American Bur‐reed (*Sparganium americanum*).
